# Case Report: Is the isolated bone change in advanced colorectal cancer necessarily malignant metastasis?

**DOI:** 10.3389/fonc.2025.1582022

**Published:** 2025-07-29

**Authors:** Huimin Xue, Xin Sun, Xiaomei Yang, Wenjuan Chen, Juan Chen, Xiujuan Qu, Ying Chen, Jinglei Qu

**Affiliations:** ^1^ Department of Medical Oncology, The First Hospital of China Medical University, Shenyang, China; ^2^ Key Laboratory of Anticancer Drugs and Biotherapy of Liaoning Province, The First Hospital of China Medical University, Shenyang, China; ^3^ Liaoning Province Clinical Research Center for Cancer, The First Hospital of China Medical University, Shenyang, China; ^4^ Key Laboratory of Precision Diagnosis and Treatment of Gastrointestinal Tumors, Ministry of Education, The First Hospital of China Medical University, Shenyang, China

**Keywords:** radiation osteitis, radiotherapy, pelvis, bone metastasis, case report

## Abstract

**Background:**

Radiation osteitis (RO) is a bone-related complication following radiotherapy (RT), often characterized by atypical imaging features. It is challenging to distinguish RO from early bone metastasis (BM), potentially leading to inappropriate treatment. Therefore, establishing reliable diagnostic criteria for accurately identifying RO is essential for improving treatment outcomes in advanced colorectal cancer (CRC).

**Case description:**

Two cases of advanced CRC patients with atypical isolated bone changes on imaging are presented. Both patients received standard chemotherapy and radiotherapy after surgery. Through comprehensive imaging studies, laboratory evaluations, and multidisciplinary team (MDT) consultations, the diagnosis of RO was confirmed instead of BM, thereby avoiding the need for an invasive pathological biopsy.

**Conclusions:**

This case report highlights imaging features of RO and provides valuable insights into differentiating RO from BM by integrating medical history, laboratory findings, and imaging results.

## 1 Introduction

Bone metastasis is one of the most common and serious bone complications in malignant solid tumors. The incidence of BM is about 3% to 7% in advanced CRC (1, 2). Nowadays, BM is mainly diagnosed by biochemical markers and bone imaging techniques, such as emission computed tomography (ECT), magnetic resonance imaging (MRI), positron emission tomography (PET), and computed tomography (CT) (3–5).

RO is often considered as the early stage of radiation-induced reactions after radiotherapy, which might develop into osteoradionecrosis or bone fracture in the future. Risk factors include the age and health status of the patient, absorbed dose, size of the radiation field, beam energy and fractionation, and so on (6, 7). It is an inflammatory reaction with a bone marrow oedema pattern, without morphological evidence of necrosis in punctuated or confluent hyperintensities on T1-weighted imaging with contrast and turbo-inversion recovery magnitude sequences (6). In pelvic radiotherapy, RO of sacral bone is the most common manifestation due to the amount of red bone marrow within the central field of pelvic radiation (6). However, there is little evidence for the diagnosis of RO in the pelvis and it is easily misdiagnosed as BM, resulting in unfavorable outcomes.

Herein, we reported two advanced CRC patients with isolated bone change, albeit finally confirmed as RO rather than BM; we have also presented here a review of the diagnosis and the potential differential diagnosis. This manuscript is written following the *CARE checklist* (8).

## 2 Case presentation

**Figure 1:**
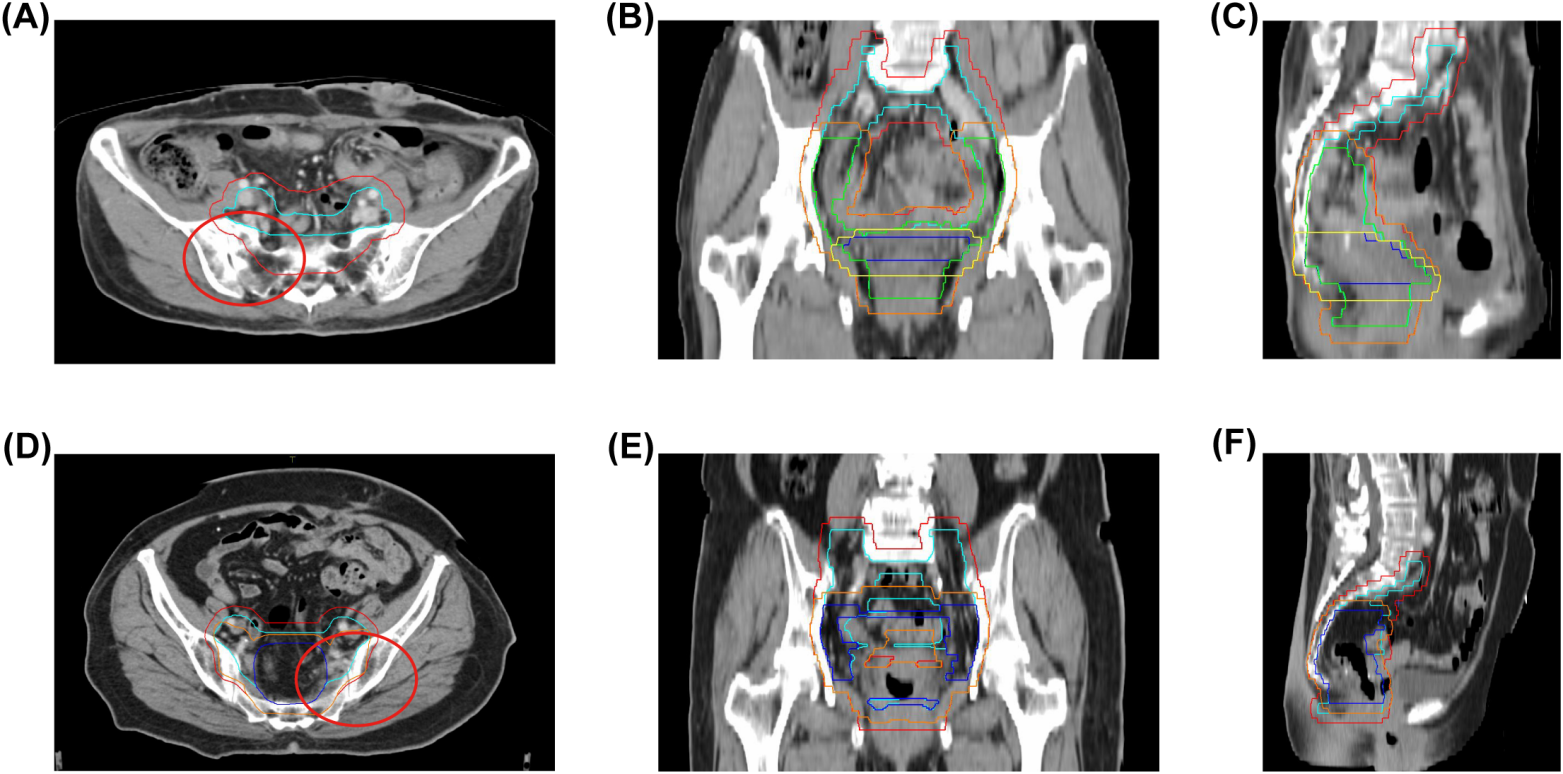
Radiation therapy target volumes and bone coverage. **(A–C)** Case 1: Clinical target volumes (CTVs) highlighted in light blue (CTV1), green (CTV2), and dark blue (CTV3); Planning target volumes (PTVs) in red (PTV1), orange (PTV2), and yellow (PTV3); radiation field includes sacrum and iliac bones. **(D–F)** Case 2: CTVs in light blue (CTV1) and dark blue (CTV2); PTVs in red (PTV1) and orange (PTV2); radiation field includes sacrum and iliac bones. Red circles denote regions of subsequent bone change in both cases. CTV, Clinical target volume; PTV, Planning target volumes.

**Figure 2:**
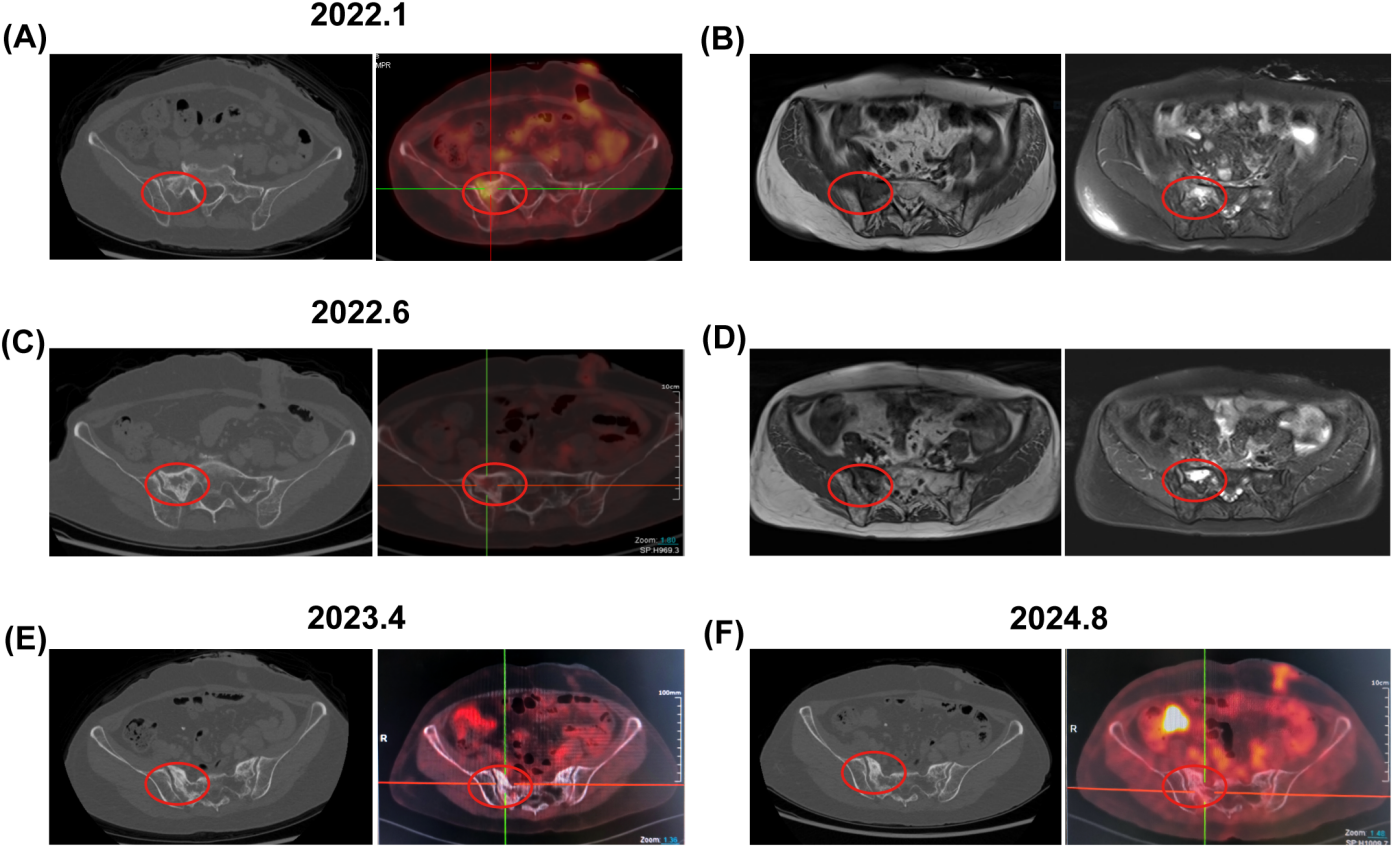
Bone changes observed in the patient from case 1. January 2022: **(A)** Enhanced CT and PET-CT images revealed low-density bone shadows in the sacrum, associated with increased metabolic activity (SUV 3.3). **(B)** An axial T1-weighted MRI image displayed low signal intensity in the right sacrum, while axial and coronal fat-suppressed T2-weighted images indicated patchy long T2 signals, suggestive of edema or infiltrative changes. June 2022: **(C)** Enhanced CT and PET-CT images continued to show persistent low-density bone shadows in the sacrum, albeit with decreased metabolic activity (SUV 2.8). **(D)** MRI findings mirrored previous observations, with low signal intensity in the right sacrum and patchy long T2 signals on axial and coronal fat-suppressed T2-weighted images. April 2023 and August 2024: **(E, F)** PET-CT scans revealed changes in the sacrum with no metabolic increase. The red circle highlights the area of bone change. CT, computed tomography; PET-CT, positron emission tomography-computed tomography; MRI, magnetic resonance imaging.

Case 1: Background: In March 2020, a 57-year-old female patient presented with diarrhea (**Table 1**). The patient reported no personal or familial history of cancer. A colonoscopy revealed an ulcerative mass in the sigmoid colon. Post-surgical pathology revealed the mass as a moderately differentiated tubular adenocarcinoma, with pathological staging classified as pT4bN0M0 according to the AJCC Version 8.0 criteria. Immunohistochemical analysis indicated microsatellite stability (MSS), and genetic testing revealed somatic mutations in KRAS, specifically p.G12V. Treatment history: Postoperatively, the patient received two cycles of CapeOX (capecitabine and oxaliplatin). Follow-up CT and PET/CT in September 2020 showed no signs of recurrence or metastasis. The patient subsequently underwent radiotherapy from September to October 2020, receiving a total dose of 50.4 Gray (Gy) over 28 fractions, without concurrent chemotherapy due to myelosuppression. The region of radiotherapy is shown in **Figures 1A–C**. Following radiotherapy, the patient continued with five cycles of capecitabine until May 2021. Imaging findings: However, on January 5, 2022, an enhanced CT scan identified a metastatic node in the peritoneal cavity (**Supplementary Figure S1A**). The patient’s disease-free survival (DFS) was 20 months. PET/CT imaging revealed a maximum standardized uptake value (SUVmax) of 14.6 for the node, alongside bone abnormalities. Specifically, the SUVmax for the sacral bone was measured at 3.3 (**Figure 2A**). Enhanced MRI of the pelvic cavity demonstrated low-intensity signals on T1-weighted imaging and high-intensity signals on T2-weighted imaging in the bilateral sacrum and left ilium (**Figure 2B**). Gadolinium-DTPA-enhanced T1-weighted MRI indicated inhomogeneous enhancement of the bilateral sacroiliac joint, with a blurred joint space and no significant swelling of the surrounding soft tissue. Concurrently, the patient’s alkaline phosphatase (ALP) level was 107 U/L (**Supplementary Table S1**), and the patient exhibited no bone-related symptoms. The possibility of bone metastases could not be excluded. From January 2022 to June 2022, the patient underwent one cycle of treatment with XELIRI (capecitabine/Irinotecan) and bevacizumab monoclonal antibody. Due to Grade-3 vomiting and Grade-4 myelosuppression, the treatment regimen was adjusted to CapeOX and bevacizumab monoclonal antibody for nine cycles. Additionally, zoledronic acid was administered over six cycles. During the treatment period, there was a reduction in the size of the metastatic node, accompanied by a decrease in SUVmax to 10.6 (**Supplementary Figure S1B**). The SUVmax of the sacrum was recorded at 2.8, with no additional improvements observed (**Figure 2C**). MRI scans indicated no significant changes compared to previous assessments (**Figure 2D**), and ALP levels remained within normal limits at 82 U/L. Outcomes: We evaluated the possibility of surgical or radiotherapeutic intervention for the metastatic node located in the peritoneal cavity pelvic metastasis. The MDT concluded that the patient had a history of rectal malignant tumor. Twenty-one months after surgery, imaging examination during follow-up revealed new nodular shadows in the ileal bowel. Comparison with previous images suggested metastasis. After receiving chemotherapy combined with targeted therapy, the lesions showed regression, and tumor markers returned to normal, indicating the effectiveness of the treatment. Regarding the sacral lesion, it is located within the radiotherapy target area, presenting as diffuse patchy changes without osteolytic changes. Imaging observation showed no significant progression, and radiation osteitis cannot be excluded. We evaluated the diagnosis of bone changes and the possibility of surgical intervention for metastatic nodules located in the abdominal cavity. The MDT, composed of experts in radiation therapy, radiology, pathology, oncology, and surgery, reviewed the case. The radiation oncologist noted that the bone changes occurred 16 months after radiotherapy, in an area consistent with the radiation field, conforming to the conditions for RO. The radiologist emphasized that the sacral lesion showed diffuse patchy changes on CT/MRI without osteolytic destruction, low metabolic activity on PET-CT scan, and remained stable during follow-up without progressive enhancement or soft tissue mass formation, which was inconsistent with typical bone metastasis imaging. The ileocecal nodule was considered metastatic. The pathologist believed that a sacral biopsy was needed to clarify the nature of the lesion, but the patient had a history of myelosuppression from previous chemo-radiotherapy, making the biopsy risky. The surgeon stated that for the paraintestinal nodule, if the patient was proactive about treatment, abdominal exploration could be performed, and surgical resection could be considered if there were no other metastases. Summarizing the above opinions, the oncologist pointed out that the patient had a history of rectal malignant tumor surgery, followed by ileocecal metastasis that shrank after chemotherapy and targeted therapy, with normal tumor markers indicating effective treatment. For the sacral changes, the patient had no obvious symptoms, normal ALP, and the lesion was within the previous radiation field. The team unanimously agreed that the possibility of metastatic disease was extremely low. Due to the patient’s refusal of subsequent MRI examinations, we used PET-CT for follow-up. The patient’s PET-CT scans on April 2023 and August 2024 showed sacral changes without metabolic elevation (**Figures 2E, F**), and the patient remains alive, which further confirms the diagnosis of RO.

**Table 1:** Summary of the characteristics and treatments of the two cases.

Patients	1	2
Age	57	64
Sex	female	female
Race	Asian	Asian
Site of the cancer	sigmoid	rectum
Staging after surgery	pT4bN0M0	pT4bN0M0
Radiotherapy	50.4 Gy in28 fractions	50.4 Gy in28 fractions
Site of metastasis	peritoneal cavity	left adnexal area
Site of RO	Sacrum, ilium	Sacrum, ilium
MMR protein analysis	MSS	MSS
Gene test	somatic mutations of KRAS, FGFR and PIK3CA	somatic mutations of KRAS

**Figure 3:**
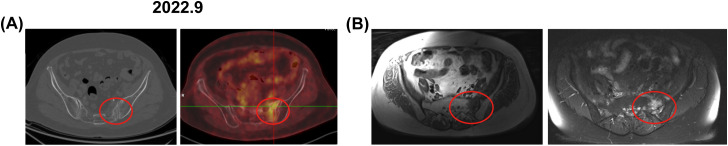
Bone changes observed in the patient from case 2. September 2022: **(A)** Enhanced CT and PET-CT images revealed increased metabolic activity in the left sacrum, with a SUV of 4.6. **(B)** An axial T1-weighted MRI image displayed low signal intensity in the left sacrum, axial and coronal fat-suppressed T2-weighted images exhibited patchy long T2 signals. The red circle marks the area of bone change. CT, computed tomography; PET-CT, positron emission tomography-computed tomography; MRI, magnetic resonance imaging.

Case 2: Background: In December 2020, a 64-year-old female patient presented with hematochezia (**Table 1**). The patient denied any medical history or family history of cancer. A colonoscopy identified a rectal mass, and subsequent surgical pathology confirmed a diagnosis of moderately differentiated, partially ulcerative tubular adenocarcinoma. The pathological staging was determined to be pT4bN0M0, in accordance with the AJCC 8th Edition guidelines. Immunohistochemical analysis demonstrated microsatellite stability, and genetic testing identified somatic mutations in the KRAS gene. Treatment history: Postoperatively, the patient received two cycles of CapeOX chemotherapy, followed by radiotherapy from June to July 2021, delivering a total dose of 50.4 Gy in 28 fractions, concurrently with oral capecitabine. The region of radiotherapy is shown in **Figures 1D–F**). Following radiotherapy, the patient completed an additional three cycles of CapeOX, concluding in September 2021. Imaging findings: In March 2022, a left adnexal mass was detected, with PET/CT revealing an SUVmax of 10.9 (**Supplementary Figure S2A**). PET-CT showed no significant changes in the sacrum (**Supplementary Figure S2B**). Concurrently, carcinoembryonic antigen (CEA) levels were elevated to 25.16 ng/mL, and ALP was measured at 95 U/L (**Supplementary Table S2**), with no other abnormalities noted. The DFS period was 12 months. The patient underwent a treatment involving irinotecan and raltitrexed over eight cycles from March 2022 to September 2022. CEA levels decreased from 25.16 ng/ml to 9.42 ng/ml, and there was a reduction in the left adnexal mass, with a SUVmax of 3.7 (**Supplementary Figure S2C**). At this time, the patients had the opportunity to undergo pelvic metastases resection. However, in routine preoperative examinations, PET/CT indicated that the SUVmax in the left sacral was 4.6 **Figure 3A**. MRI showed low-intensity images on T1-weighted imaging and high-intensity images on T2-weighted imaging in sacrum and bilateral iliac bone (**Figure 3B**). ALP was 95 U/L. The patient was asymptomatic. Outcomes: The MDT evaluated the sacral lesion. The radiation oncologist noted that sacral changes occurring 15 months after radiotherapy and located within the radiation field were consistent with RO. The radiologist reviewed CT scans during treatment, revealing gradual bony changes in the sacrum, while MRI and PET-CT findings were inconsistent with typical BM. The pathologist deemed the patient unfit for sacral biopsy due to physical condition. The oncologist summarized that the patient showed a significant decrease in CEA, reduction in adnexal mass, stable ALP level, and no bone-related symptoms during treatment, concluding that chemotherapy-resistant bone metastasis was unlikely and there were no features suggestive of infection or primary bone tumor. Consequently, the patient underwent resection of the left adnexal metastatic node and remained alive with no evidence of osseous progression on follow-up.

## 3 Discussion

RO, alongside osteoradionecrosis (ORN) and pathologic insufficiency fractures (PIF), represents a spectrum of bone radiation-induced reactions (RIR) following pelvic radiotherapy (6). The radiological features of RO can overlap with those of BM or radiation-induced sarcoma, complicating diagnosis and potentially affecting patient outcomes.

The pathogenesis of RO is linked to ionizing radiation, which induces osteoblast apoptosis and enhances osteoclast activity, while also damaging hematopoietic cells in the bone marrow. This leads to reduced local blood supply, fat cell infiltration, and eventual fatty replacement of the marrow (9). The sacrum, rich in red bone marrow and centrally located in the radiation field, is particularly vulnerable (10).

Compared with RO, BM usually means a case far from no evidence of disease (NED) and is associated with poor survival outcomes (11). BM in rectal cancer patients often presents with severe pain, pathological fractures, and hypercalcemia, severely impacting quality of life (12). Currently, the gold standard for diagnosing BM is pathological examination; it is invasive and risky, especially in irradiated bone, where biopsy may lead to irreversible changes like osteonecrosis (10, 13). This is particularly challenging for elderly patients or those in poor physical condition. Şelaru et al. found that the common metastatic types in BM include moderately differentiated adenocarcinoma with abundant mucin and poorly differentiated adenocarcinoma with a very high Ki67 index (almost 100%) (14). Imaging, particularly MRI, is non-invasive and highly sensitive for early bone metastasis (15). BM from rectal cancer is predominantly osteolytic, appearing as focal mass lesions with low T1-signals due to the replacement of normal fatty marrow by malignant cells; on a T2-weighted sequence, BM usually present T2 hyperintensity due to the elevated water content and gadolinium enhancement due to increased vascularity associated therewith (16). Biochemical markers contribute to diagnosis of BM usually include tumor biomarkers, ALP, N- terminal propeptide of procollagen type I (PINP), C-terminal propeptide of procollagen type I (PICP), tartrate resistant acid phosphatase (TRAP), bone sialoprotein (BSP), and so on (17).

Diagnosing RO requires a history of pelvic irradiation. RO is featured by mottled areas of bone with osteopenia, coarse trabeculation, and areas of focally increased bone density. The changes usually begin on the iliac side of the sacroiliac joint and process to involve the entire joint (18). Histological examination revealed characteristic features including marked osteocyte destruction, absence of osteoblasts along the bone margins, lack of new osteoid tissue formation, and atrophic changes in bone tissue like the atrophic changes observed in skin or mucosal tissues (10). In CT imaging, early manifestations include decreased bone density, mild blurring of bone trabeculae, and patchy sclerosis. In the late stage, increased density in the bone marrow cavity, irregular thickening of bone trabeculae, and sclerosis of the articular surface can be observed. Therefore, sclerotic or mixed lesions within the radiation field, in the absence of soft tissue masses, suggest RO (10). Yoshioka et al. found that MRI signal patterns in RO include low signal intensity on T1-weighted images, mixed signal intensity in peripheral areas on T2-weighted images, indicating edematous changes, and gadolinium enhancement may be seen due to fibrotic changes (19). This bone marrow edema is usually diffuse and ill-defined, distinguishing it from the clear-edged edema seen in BM (20). Meixel et al. defined a RISC (Radiation-Induced Sacral Change) classification according to MRI, in which RO showed iso-/hypointense responses in T1wi, hyperintense responses in T2wi, hyperintense (punctuated or confluent) responses in T1wi +contrast, and hyperintense response in turbo-inversion recovery magnitude (6). Radiotherapy induces the migration of inflammatory cells, resulting in mild FDG uptake at the site of RO on PET/CT. In the absence of other lesions, such as osteomyelitis or malignancies, this uptake diminishes as inflammatory cells disperse from the fracture site, a phenomenon observed in our case (21). However, due to the low soft tissue resolution of CT, PET/CT exhibits limited sensitivity for detecting mild exudative lesions (22).

Both cases had metastatic nodules in the abdomen or pelvis, contradicting Xu et al.’s criterion for diagnosing ORN, which requires no recurrent tumor (10). This complex situation poses challenges for diagnosing changes in the bone. We used a multidisciplinary team (MDT) approach to diagnose RO (23). In Case 1, PET/CT showed the abnormal uptake of sacrum, which usually implies bone metastasis. Although manifestations in MRI/CT implied that the bone changes were unilateral with cortical destruction therein, there were no significant changes after anti-tumor therapy and anti-osteoporosis. We finally diagnosed the patient as solitary metastasis of peritoneal cavity with RO. In Case 2, during the anti-tumor treatment, PET/CT exhibited the abnormal uptake of sacrum. This was confused when trying to confirm the bone change was BM or some other cause. However, the bone changes in MRI/CT showed bilateral and complete cortical bone. The patient was diagnosed with solitary metastasis in the left accessory area with RO and underwent surgical removal of the solitary metastasis.

Emerging imaging techniques show promise in differentiating bone marrow changes post-radiotherapy. Diffusion-weighted imaging (DWI) may outperform conventional MRI in diagnosing PIF (24), while dynamic contrast-enhanced MRI (DCE-MRI) can assess bone marrow microcirculation to evaluate hematopoietic alterations (25). PET-MRI has demonstrated earlier PIF detection than PET-CT in cervical cancer patients (26). However, clinical implementation faces feasibility challenges. DWI requires optimized protocols to mitigate bony susceptibility artifacts, DCE-MRI necessitates contrast administration and complex pharmacokinetic modeling, PET/MRI remains limited by cost, availability, and longer acquisition times in routine practice. Future research should aim to identify biomarkers that distinguish RO from BM. Standardizing imaging and pathological protocols with advanced techniques is essential. Promoting multidisciplinary collaboration will aid in understanding diagnostic challenges.

In the case reports, the highlight was that provide valued experience for differentiate RO from BM correctly in advanced CRC patients. Although some specific laboratory tests for bone have been absent and increased CEA was not sufficiently valuable to indicate changes in bone because of the solitary metastasis of other part, we found the ALP was normal during treatment. Radiology, especially MRI, was considered as the best technique with which to diagnose RO. The study’s limitation was the lack of pathological biopsy to obtain histological evidence, which may have affected the certainty of the diagnosis. Nevertheless, the significant advantage of MDT in avoiding unnecessary invasive examinations provides valuable practical experience for clinical management of similar complex cases.

## 4 Conclusions

In conclusion, our cases indicated that isolated bone change in advanced CRC patients may be RO rather than BM. Medical history, laboratory tests, and imaging should be considered fully. However, more specific standards for differential diagnosis are warranted in future.

## Data Availability

The original contributions presented in the study are included in the article/**Supplementary Material**. Further inquiries can be directed to the corresponding authors.
