# Supplementary material for: Case Report: Is the isolated bone change in advanced colorectal cancer necessarily malignant metastasis?
Source: Front Oncol. 2025 Jul 29;15:1582022. doi: 10.3389/fonc.2025.1582022 (PMC12339342; doi:10.3389/fonc.2025.1582022)
Supplement: Supplementary file 2 [file DataSheet2.docx]

Supplementary Material

# Supplementary Figures and Tables

## Supplementary Tables

## Supplementary Table 1. The trends of laboratory indicators during the treatment of case 1.

|  | 2020.10.30 | 2021.1.27 | 4.25 | 7.26 | 8.24 | 10.13 | 2022.1.4 | 2.10 | 2.28 | 3.6 | 5.9 | 5.27 | 6.5 | 6.23 | 7.18 |
| --- | --- | --- | --- | --- | --- | --- | --- | --- | --- | --- | --- | --- | --- | --- | --- |
| Ca | 2.28 | 2.27 | 2.19 | 2.26 | **1.46↓** | 2.23 | 2.39 | 2.2 | 2.18 | **2.03↓** | **2.03↓** | 2.18 | **1.97↓** | 2.28 | 2.42 |
| P | 1.24 | 1.44 | 1.3 | 1.29 | 0.89 | 1.35 | 1.38 | 1.16 | 1.27 | **0.8↓** | 0.89 | 1.28 | 0.93 | 1.24 | 1.47 |
| ALP | 71 | 87 | **115↑** | 92 | **122↑** | **106↑** | **107↑** | 83 | **121↑** | **128↑** | 63 | 82 | **103↑** | 100 | **112↑** |
| CEA | 2.19 | 2.84 | 3.44 | **8.61↑** | **6.14↑** | 3.68 | 3.09 | **7.8↑** | **4.87↑** |  | 2.79 | 3.24 |  | 3.85 | 3.22 |
| CA19-9 | 1.37 | 1.21 | 1.32 | <0.60 |  | <0.60 | <0.60 | 0.62 | <0.60 |  | 0.69 | <0.60 |  | 0.61 | <0.60 |

Ca: calcium, mmol/L; P: phosphorus, mmol/L. ALP, alkaline phosphatase, U/L; CEA, carcinoembryonic antigen, ng/mL; CA19-9, Carbohydrate Antigen 19-9, U/mL.

**Supplementary Table 2.** The trends of laboratory indicators during the treatment of case 2.

|  | 2021.8.24 | 2022.3.2 | 2022.5.2 | 2022.6.28 | 2022.8.24 |
| --- | --- | --- | --- | --- | --- |
| Ca | 2.22 | 2.29 | 2.34 | 2.25 | 2.26 |
| P | **1.54↑** | **1.58↑** | 1.43 | 1.3 | 1.22 |
| ALP | 70 | 95 | 83 | 94 | 92 |
| CEA | 1.47 | **25.16↑** | **5.4↑** | 4.01 | **9.42↑** |
| CA19-9 | 20.6 | **83.37↑** | 24.34 | 18.77 | 24.41 |

Ca: calcium, mmol/L; P: phosphorus, mmol/L. ALP, alkaline phosphatase, U/L; CEA, carcinoembryonic antigen, ng/mL; CA19-9, Carbohydrate Antigen 19-9, U/mL.

## Supplementary Figures


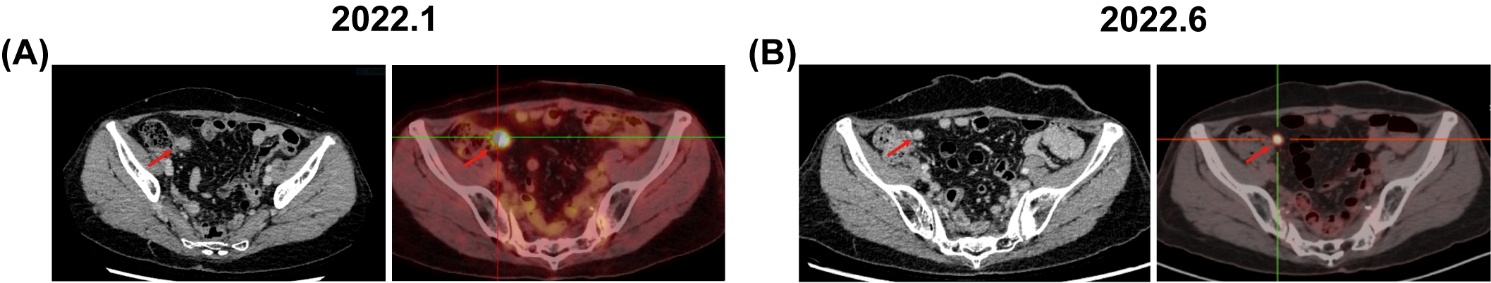


**Supplementary Figure 1.** Metastatic nodules of the patient in case 1. January 2022: (**A**) Enhanced CT and PET-CT images showed a nodule beside the ileocecal intestine (SUV 14.6). June 2022: (**B**) Enhanced CT and PET-CT images showed a reduced nodule beside the ileocecal intestine compared to before (SUV 10.6). The red arrow presented the solitary metastatic node in left adnexal area. CT, computed tomography; PET-CT, positron emission tomography-CT.

**
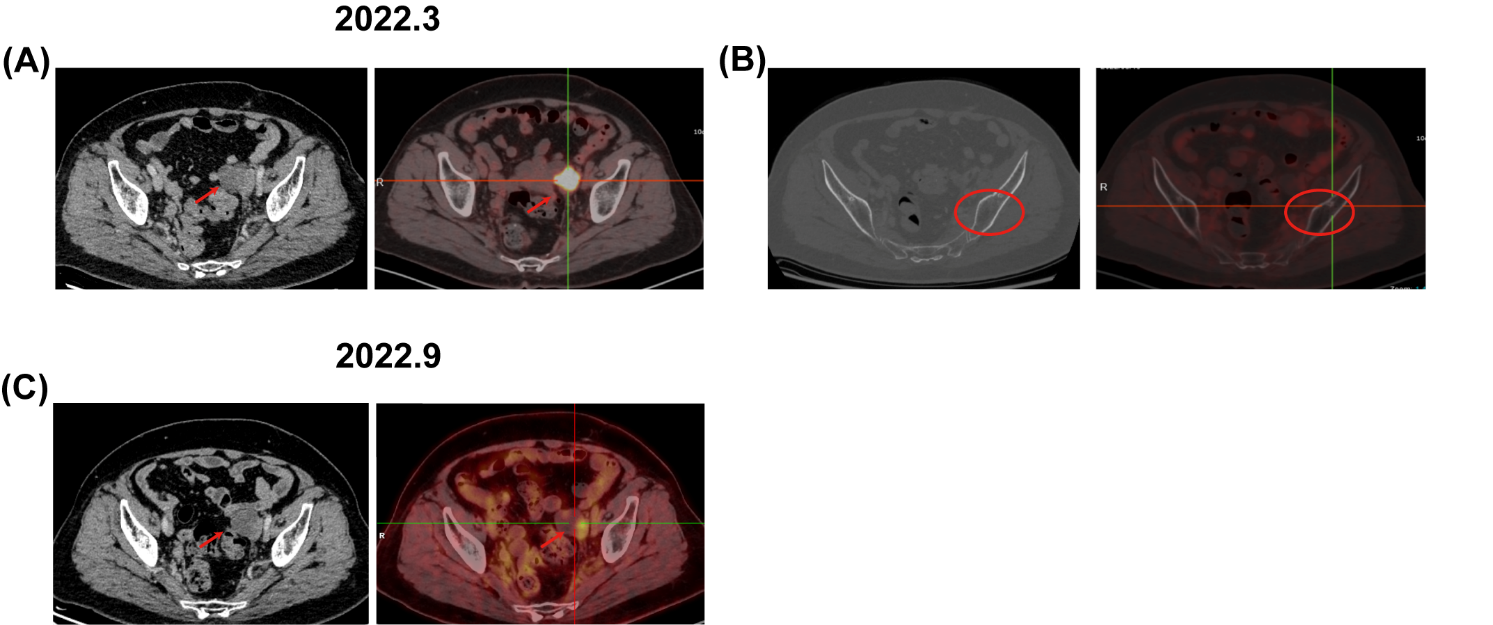
**

**Supplementary Figure 2.** Metastatic nodules of the patient in case 2. March 2022: (**A**) Enhanced CT and PET-CT showed a nodule in the left adnexal area (SUV 10.9); (**B**) enhanced CT and PET-CT showed no obvious abnormalities in the sacrum. September 2022: (**C**) Enhanced CT and PET-CT showed reduced uptake in the left adnexal area (SUV 3.7). The red arrow presented the solitary metastatic node in left adnexal area. The red circle marked the bone change. CT, computed tomography; PET-CT, positron emission tomography-CT.
